# Comparison of typical Thai and Hungarian personality profiles using the Zuckerman–Kuhlman–Aluja Personality Questionnaire

**DOI:** 10.1038/s41598-023-40654-z

**Published:** 2023-08-19

**Authors:** Zsuzsanna Kövi, Tinakon Wongpakaran, Nahathai Wongpakaran, Virág Zábó, Béla Birkás, Zsuzsanna Mirnics

**Affiliations:** 1 Centre of Specialist Postgraduate Programmes in Psychology, Institute of Psychology, Károli Gáspár University of the Reformed Church, Budapest, 1037 Hungary; 2 Department of Psychiatry, Faculty of Medicine, Chiang Mai University, 110 Intawarorot Rd., T. Sriphoom, A. Meung Chiang Mai, Chiang Mai, 50200 Thailand; 3 Doctoral School of Psychology, ELTE Eötvös Loránd University, Budapest, 1075 Hungary; 4 Institute of Psychology, Eötvös Loránd University, Budapest, 1075 Hungary; 5 Institute of Behavioural Sciences, Medical School, University of Pécs, Pécs, 7624 Hungary; 6 Department of Personality and Health Psychology, Institute of Psychology, Károli Gáspár University of the Reformed Church, Budapest, 1037 Hungary

**Keywords:** Psychology, Health care

## Abstract

The aim of our study was to compare typical Thai and Hungarian personality profiles of the Zuckerman–Kuhlman–Aluja Personality Questionnaire (ZKA-PQ). 672 Thai and 647 Hungarian were included in our study. The distribution of age, gender and education level were matched. The ZKA-PQ was administered that measures Aggression, Extraversion, Activity, Sensation Seeking and Neuroticism. We tested reliability, the structural invariance and analyzed aggregated mean profiles for cultures as well as typical profiles by cluster analyses. Reliability of factors were acceptable in both cultures, but some facets (especially AC3 Restlessness) showed low reliability. The global Tucker’s coefficient of congruence (TCC) for cross-cultural factorial invariance was 95. We have also run a Multigroup Confirmatory Factor Analysis, but fit indices were not adequate. Cross-cultural neural network invariance was not met either. Hungarians scored significantly higher on Extraversion, Sensation Seeking, Aggression and Activity. Cluster-analyses revealed six typical profiles: Introverted impulsive, Reserved, Resilients, Overcontrolled, Aggressive impulsive and Positive sensation seeker. Majority of first two clusters were Thai respondents, majority for last two clusters were Hungarians. In sum, there were some cross-cultural congruence in factor structure, but strict invariance was not fulfilled. Comparison of mean profiles remain tentative, but cluster analysis revealed cross-cultural differences in typical profiles.

## Introduction

Over the past several decades, trait psychology has emerged as the theoretical basis of individual differences in personality^1^, mostly based on factor-analytic approach, which regard personality factors as basic personality dimensions. International comparative studies utilizing validated questionnaires have portrayed that personality traits are in many respects universal^2^. Zuckerman and colleagues^3^ aimed to create a culture-invariant personality model (AFFM, Alternative Five Factor Model) and proposed that those factors should be included in such a model, which have biological-genetical bases. Rolland^4^ concluded that cross-cultural stability of the factorial structure stands for the evidence of the identification of human universals. The use of strategies of maximazing factorial invariance (fit with a pre-existing model) has found to be a useful step in establishing universal factors, however, it inhibits the chance of learning about interesting and relevant discrepancies that relate to cultural differences^5^.

Given the relatively strong relations of personality factors to biological variables, some researchers refer AFFM as psychobiological model of personality^6–8^. However, it should be noted that psychobiological personality model is more often associated with the contributions of C. Robert Cloninger^9^ and his temperament and character inventory (TCI), which not only measures bio-psycho-social aspects of personality, but also incorporates spiritual aspects, such as self-transcendence. It is notable, that a series of recent researches of Cloninger et al. focuses on new molecular and complex genetical findings of human temperament^10–12^.

Regarding AFFM, research in the last decade focused largely on the validity and cross-cultural invariance of a new factor-facet version questionnaire, Zuckerman–Kuhlman–Aluja Personality Questionnaire (ZKA-PQ)^13–15^ and its shortened form^16^ (ZKA-PQ-SF). These questionnaires (ZKA-PQ and ZKA-PQ/SF) have received attention in genetical research^17–19^ and in psychiatric or clinical settings^20–22^ in the past decade.

The latest version of the full-lenght questionnaire (ZKA-PQ)^13^ was also found to be a useful tool in clinical practice to aid the psychological explanation and the diagnosis of personality disorders^21,23^. Neuroticism was linked to most personality disorder scales, aggressiveness and sensation seeking correlated with antisocial personality disorder, and extraversion negatively correlated with avoidant and dependent personality disorders. All these studies demonstrate the utility and validity of the factor-analytic personality approach in clinical settings.

Although the reliability and validity of ZKA-PQ has been confirmed through all these studies, cross-cultural research on ZKA-PQ showed that cross-cultural differences in mean profiles were weak^14^. Further, it was noted that scalar invariance was not met and therefore mean profiles comparisons remained tentative. This is in line with other cross-cultural research results with other Five-Factor Model questionnaires, which questioned the validity of mean personality profile comparison of different cultures^24–26^.

However, some other research results^27,28^ provided evidence for meaningful cross-cultural comparisons of national average profiles of personality factors (Big Five factors). Research found that countries with similar profiles on a multidimensional scaling map were also geographically located close to each other in reality^29^.

Previously reported Western-Eastern cultural studies^24^ have found that main difference between the individuals of Western-Eastern cultures lies in their level of Extraversion. McCrae^27^ found negative correlation between extraversion and collectivism.

**Figure 1:**
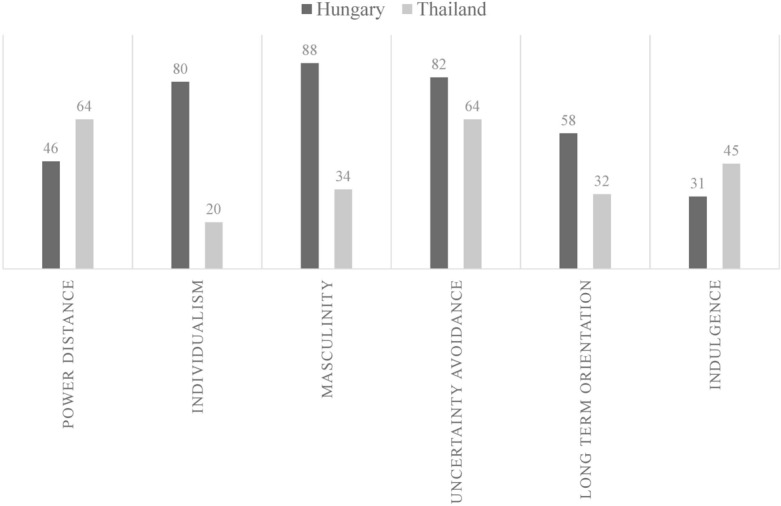
Hofstede indices for Thailand and Hungary.

It is notable that Hungary is regarded as a rather individualistic, masculine society, wheras Thailand is a rather collectivistic, feminine culture, according to Hofstede indices^30^. This means that Thai people, generally take more responsibility for others and tend to be less assertive and competitive than ones in individualistic and masculine society (e.g. Hungary). Thai and Hungarian Hofstede scores can be found on Fig. 1.

In the middle of the last century, Blanchard^31^ has pointed out that typically Thai individuals are characterized by low sensation seeking and low restlesness. "*There is no doubt that moderation and peacefulness are among the most important Thai social values*" (p. 482). A research examining Thai students, found that Thai students have lower scores on the Sensation-Seeking Scale (SSS) than American students and Buddhist monks have even lower SSS scores than Thai students^32^. However, it has to noted that these researches were done more than fifty years ago. Another characteristic of Thai culture, as being a Buddhist country, is that it strongly prohibits against physical aggression and rule breaking. However, in a cross-cultural comparison, Thai undergraduate students were as aggressive as Indonesian and Australian students^33^.

These cross-cultural studies utilized the statistical methodology of comparing aggregate mean scores. However, aggregation of scores has been criticized by some person-oriented researchers. For example, Bergman and colleagues^34^ have questioned the frequently applied assumption of the variable-oriented factor-analytic approach, namely, that the interrelations between variables are the same for all individuals. Bergman and colleagues^34^ suggest the application of person-oriented approach, namely the use of cluster analyses to unfold the typical holistic patterns and to form subgroups within the samples.

Distinguishing the “variable-” and “person-oriented approach” within personality psychology originates from Jack Block^35^ who defined the former as a methodology focusing on relations between variables, and the latter as a methodology unfolding the typical configuration of a dynamic set of variables. Thus, variable-oriented methods unfold groups of variables (factors, aggregated dimensions), whereas person-oriented analyses unfold groups of similar individuals (clusters, types). With regards to personality psychology, personality dimensions can be considered as basic units of variable-oriented approach, and personality types as units of person-oriented approach.

It must be noted, that some variable-oriented methods may apply pattern analyses and meet the interactionist, dynamic and holistic principles of the person-oriented approach (such as growth mixture models, dynamic factor analysis). Additionally, pattern analyses may involve patterns of covariations as well as typical configurations of distinct scores. Thus, the approaches overlap to some extent and the integration of these different methodologies is progressively emphasized^36^. Laursen and Hoff^36^ highlight that combination of person-oriented and variable-oriented approaches can lead to a deeper understanding of the processes and patterns of human development.

Regarding the ZKA-PQ, a multicultural (including 22 cultures) study^37^ have examined so far the typical profiles and identified five cross-culturally stable profiles: resilients (high extraversion and low neuroticism), overcontrolled (low extraversion and high neuroticism), undercontrolled (high aggression and high sensation seeking), reserved (low aggression and low sensation seeking) along with an ordinary (average) cluster.

### Aims of the study

The overarching aim of our study was to compare Thai-Hungarian AFFM personality profiles both with variable- and person-oriented methodologies. The aim was to compare two highly different cultures with such a questionnaire that provides measuring universal personality factors.

To our present knowledge, the Zuckerman–Kuhlman–Aluja Personality Questionnaire has not been validated in South-Eastern Asian countries and few cross-cultural personality questionnaire researches have been conducted with regards to Thai people, especially in relation to an individualistic European country, such as Hungary is.

We chose the AFFM model, and its Zuckerman–Kuhlman–Aluja Questionnaire (ZKA-PQ) for our cross-cultural study as it aims to measure universal basic personality dimensions.. We also aimed to provide Thai validation of the questionnaire. There has been a multicultural validation study on ZKA-PQ clusters with already 22 countries involved^15^, including Hungary, but not including Thailand.

Thus, as first step, we aimed to test the reliability of Thai version. Second, we aimed to test the cross-cultural invariance of the factor-analytic model as well as the relation of facets (subfactors). At last, we conducted analyses for our main aim (comparison of Hungarian and Thai personality profiles) with two different methodologies: 1) comparison of average factor and facet scores, 2) examining the cultural distributions of typical profiles (clusters).

### Hypotheses

As far as our present knowledge, no previous study has investigated Thai-Hungarian comparison in light of AFFM model so far. Based on above presented researches, we hypothetized:

1. Factors of Zuckerman–Kuhlman–Aluja Personality Questionnaire show reliability across cultures (as shown by previous cross-cultural studies^15^).
2. Cross-cultural scalar invariance will not be met (as shown by previous cross-cultural studies^15^).
3. We expect only negliagable differences in mean personality^15^ across cultures, with higher scores in Extraversion and Sensation Seeking for Hungarians (based on previous Western-Eastern comparisons^24,31^ and based on results that extraversion is negatively linked to collectivism^27^)
4. We also hypothesize that cluster analytic typical profiles will correspond to previously identified typical profiles in cross-cultural research of 22 countries^37^ (namely, overcontrolled, undercontrolled, resilient, reserved, ordinary).

## Materials and methods

### Participants

Altogether 1319 individuals (672 Thai and 647 Hungarian) were included in our final sample. The study was approved by Psychologics Ethics Committee of university of the first author and an independent Ethics Committee for Human Research of the university of the second author.

In Thailand, the study design involved conducting a survey among general and nonclinical participants, employing a nonsampling method. The inclusion criteria required participants to be between 18 and 59 years old, possess the ability to understand, read, and write in Thai language, be capable of utilizing an electronic questionnaire created with Google Forms, and own electronic devices such as smartphones, tablets, or notebooks that can access the researcher’s online form. The exclusion criteria consisted of individuals with a history of psychiatric disorders or those currently under the care of a psychiatrist, as well as individuals diagnosed with or receiving treatment for substance use disorder.

In Thailand, the research was conducted in Chiang Mai, the main city in the northern region of Thailand. However, participants were not restricted to individuals residing in Chiang Mai, but rather open to individuals from any part of Thailand who had internet access and met the criteria (e.g., age 18–59). Nevertheless, it is anticipated that the majority of the participants were from Chiang Mai and nearby provinces, such as Chiang Rai and Lampang, as the invitations primarily targeted individuals in these areas.

In Hungary, in order to generate a matched sample regarding age, gender, education distributions, we took the Thai sample as the reference sample. There were two larger Hungarian samples our data originated from: one from an earlier study (see details^38^) of workers in 19 different companies from six different cities (Budapest, Paks, Vértes, Miskolc, Sülysáp, Budaörs), and another one recruited from universities of capital city, Budapest. Altogether, 3737 Hungarian individuals completed the ZKA-PQ questionnaire. 74% was living or studying in capital city of Hungary (Budapest). 22% of respondents (mainly company workers) were from county ‘Tolna’ and the remaining respondents (4%) were from other parts of Hungary (with no regional restrictions). Individuals from this larger database were randomly assigned to the final dataset, based on calculated quotas to match the gender, age and education distribution of the Thai sample.

In our final sample, the majority (73.3%) of participants were females, half of the respondents were university students (50.0%), and around half of sample was under age 26 (51.9%). 89.5% of respondents below 26 yrs were university students. Distributions are presented in Table 1. Age, gender and education distributions did not differ significantly in our final samples [Gender (Chi2 = 0.17, *p* = 0.709), Age (Chi2 = 0.78, *p* = 0.941) and Education (Chi2 = 1.35, *p* = 0.718)].

**Table 1:** Sample characteristics of Thai and Hungarian samples.

		Thai		Hungarian university students’ sample		Hungarian company’s sample		Hungary final matched sample		Total	
		N	%	N	%	N	%	N	%	N	%
Gender	Male	176	26.2%	467	21.8%	1156	71.7%	176	27.2%	352	26.7%
	Female	496	73.8%	1676	78.2%	457	28.3%	471	72.8%	967	73.3%
Age	Max 25	343	51.0%	1757	82.1%	73	4.5%	341	52.7%	684	51.9%
	26–35	205	30.5%	260	12.1%	466	28.9%	185	28.6%	390	29.6%
	36–45	59	8.8%	73	3.4%	503	31.2%	59	9.1%	118	8.9%
	46–55	43	6.4%	39	1.8%	465	28.8%	43	6.6%	86	6.5%
	Above 55	22	3.3%	11	0.5%	106	6.6%	19	2.9%	41	3.1%
Education	Max primary	19	2.8%	4	0.2%	103	6.5%	13	2.0%	32	2.4%
	Max secondary	38	5.7%	46	2.1%	681	42.7%	38	5.9%	76	5.8%
	In progress	329	49.0%	1940	90.5%	0	0.0%	330	51.0%	659	50.0%
	University/college degree	285	42.5%	153	7.1%	810	50.8%	266	41.1%	551	41.8%
	Total	672	100.0%	2143	100.0%	1594	100.0%	647	100.0%	1319	100.0%

Larger Hungarian samples were recruited from university students and companies, but quota-based random assignment was applied to create final, matched sample in order to provide matched age, gender and education distributions between Hungary and Thailand.

### Instrument

The ZKA-PQ^13^ was administered, which contains five factors with four facets per factor and with 10 items per facet: (a) AG: Aggressiveness (AG1: Physical Aggression, AG2: Verbal Aggression, AG3: Anger, and AG4: Hostility); (b) AC: Activity (AC1: Work Compulsion, AC2: General Activity, AC3: Restlessness, and AC4: Work Energy); (c) EX: Extraversion (EX1: Positive Emotions, EX2: Social Warmth, EX3: Exhibitionism, and EX4: Sociability); (d) NE: Neuroticism (NE1: Anxiety, NE2: Depression, NE3: Dependency, and NE4: Low Self-Esteem); and (e) SS: Sensation Seeking (SS1: Thrill and Adventure Seeking, SS2: Experience Seeking, SS3: Disinhibition, and SS4: Boredom Susceptibility). Responses were made using 5-point Likert type ratings. Items of the ZKA–PQ questionnaire with the scoring instruction can be found in article of Aluja et al.^13^. It is freely available for any interested researchers, except for commercial use.

### Procedure

The ZKA-PQ^13^ was administered in native language (Thai and Hungarian) of participants. In Thailand, to recruit participants, several methods were employed, including: (1) placing banners on websites to promote the study to users, (2) posting study advertisements on the Department’s Facebook page, and (3) distributing flyers within the community for advertisement purposes. Interested individuals were provided with a link to give their consent and complete the questionnaires, which included the Thai Version of the ZKA-PQ (Thai ZKA-PQ), Core Symptom Index (CSI-15), Neuroticism Inventory-15, and demographic data. Participants received a payment of 100 baht [equivalent to 2.85 US dollars (July 2023)] for each completed set of questionnaires.

Ethical considerations were taken into account throughout the study. The invitation process was conducted without inducement or coercion. Participants’ identities were kept confidential and replaced with unique research codes. The researchers’ assistant maintained separate files for the identification and participant codes. Communication between researchers and participants occurred exclusively through the research assistant. Participants were requested to provide their contact information (e.g., email, cell phone, Line app, or any private contact) to receive notifications from the research assistant.

Hungarian participants, both company workers and university students completed an online version of the anonymous questionnaire. Hungarian participants were recruited from two different platforms. University recruitment (in capital city of Hungary) was carried out in psychology classes. Students were also encouraged to invite their acquaintances to participate in the study. However, the university-recruited sample comprised 90.5% of university undergraduate students. After participants had completed the battery, academic staff of the personality psychology classes provided an explanation of questionnaire. To encourage participation, an automatically generated report was also provided (anonymously) to all respondents including some interpretation of their ZKA-PQ outcomes. The second Hungarian data collection was completed in 19 different companies from 6 different Hungarian cities (Budapest, Paks, Vértes, Miskolc, Sülysáp, Budaörs). These companies all had their own Human Resources Department that helped organize data collection. In return, executives were offered a report on the companies’ mean personality profiles.

### Data analyses

First, we tested skewness and kurtosis of the examined scales. We applied the rule to test if these values were in the range of  − 1 and 1, as suggested by Aluja et al.^39^. Reliability of scales were assessed with Cronbach alpha values (calculated in SPSS^40^) and with Omega values (calculated in JASP programme^41^). To test the factor structure, at first, an exploratory factor analysis was carried out (Principal Axis Factoring with Varimax rotation as proposed by Aluja et al.^13^), then we tested how well the factor structure replicates the original Spanish validation model published in 2010 by Aluja^13^. The Tucker congruency coefficient^42,43^ was calculated for pairwise comparison based on the results of the original Spanish and our two samples of Hungarian and Thai participants. Then, we applied a multigroup CFA to confirm the structure of the five-factor model proposed by Aluja et al.^13^, using AMOS^44^. Different models for the 20 facets and 5 factors with additional complexity were tested (similarly as proposed by Aluja et al.^13^): at first, the simple structure (all facets were linked to their own single latent factor only) was tested, then the model^45^ including the relations by the secondary loadings above 0.30, then above 0.25. As a next step, correlated error terms were applied based on modification indices. Subsequently, three additional measurement invariance steps were carried out (1) metric (weak factorial) invariance with equivalence of factor loadings; (2) scalar (strong factorial) invariance with equivalence of item intercepts or thresholds; and (3) residual (strict or invariant uniqueness) invariance with equivalence of items’ residuals.

We also applied the following fit indices: The Tucker-Lewis index (TLI)^46,47^, the comparative fit index (CFI)^48^, and the root mean square error of approximation (RMSEA,^49,50^). A good fitting model is characterized by a χ2/df ratio less than 3^51,52^ (or less strictly below 5)^53^. CFI and TLI values close to 0.95 or greater indicate good fit, however CFI values are also considered acceptable based on the 0.90 criterion^54^. RMSEA values up to 0.05 indicate a close fit, but values up to 0.08 can be accepted as fair fit^51^. It is important tonote that cutoff values are a topic of considerable controversy^55,56^.

Then we also examined the relations of scales with a neural network analysis of JASP^41^. This network is calculated based on partial correlations between variables. The network model was selected based on the Extended Bayesian Information Criterion (EBIC)^57^ and estimated by Graphical Gaussian Models (GGM) combined with a graphical least absolute shrinkage and selection operator (LASSO) method^58^. We have applied analysis option of EBICGlasso with normalized centrality measure and tuning parameter of 0.50, using the R package ‘qgraph’ with ‘EBICglasso’ estimation^59^. Network invariance was tested by R package of ‘NetworkInvarianceTest’^60,61^.

As a next step, we applied different person-oriented methodologies, namely model-based clustering^62^ and hierarchical clustering with k-means relocation^63^. We used the ROPSTAT statistical package^64^, a general statistical package appropriate for conducting person-oriented analyses. This package implements ‘mclust’ R package for model-based clustering. We evaluated cluster solutions with different indices (for model-based clustering the BIC value^62^, for hierarchical clustering the ESS increase value and homogeneity index^63^). ESS% within-cluster homogeneity measure can be defined as follows: EESS% = 100 ∗ (SStotal − SScluster)/SStotal = 100 ∗ (1 − SScluster/SStotal). SStotal is the sum of the sum of squared deviations from the input variable means for the whole sample. SScluster is the sum of squared deviations from the input variable centroids for each cluster. The homogenity coefficient of a cluster is the average of the pairwise within-cluster distances of cases^65^.

### Ethics approval and consent to participate

The study was conducted according to the guidelines of the Declaration of Helsinki. Ethical approval was given by independent ethics committees of universities of first (ethical approval number: 289/2016/P) and second authors. Informed consent was obtained from all participants included in the study.

## Results

### Descriptive statistics

Descriptive statistics can be seen in Table 2, and reliability analyses of scales are presented in Table 3.

**Table 2:** Descriptive statistics of scales per cultures.

Scale	Thai				Hungarian			
	Mean	SD	Skewness	Kurtosis	Mean	SD	Skewness	Kurtosis
AG1 physical aggression	1.85	.52	.62	− .03	2.04	0.56	0.81	0.59
AG2 verbal aggression	2.41	.40	.29	.31	2.58	0.49	0.08	0.18
AG3 anger	2.20	.46	− .02	− .34	2.16	0.60	0.49	− 0.23
AG4 hostility	2.12	.43	.05	.03	2.05	0.47	0.29	− 0.29
SS1 thrill and adventure seeking	2.19	.51	.05	− .02	2.29	0.61	0.15	− 0.43
SS2 experience seeking	2.54	.49	.09	− .06	2.80	0.52	− 0.25	− 0.04
SS3 disinhibition	2.24	.48	.13	− .21	2.25	0.58	0.22	− 0.16
SS4 boredom susceptibility/impulsivity	1.91	.42	.67	.78	1.95	0.44	0.51	0.52
AC1 work compulsion	2.51	.47	− .08	.38	2.37	0.53	0.04	− 0.27
AC2 general activity	2.33	.46	.27	.51	2.77	0.56	− 0.16	− 0.20
AC3 restlessness	2.41	.51	.28	.05	2.39	0.54	0.40	− 0.17
AC4 work energy	3.01	.47	.00	− .45	3.20	0.54	− 0.60	0.05
EX1 positive emotions	3.02	.46	− .26	.08	3.24	0.44	− 0.60	0.59
EX2 social warmth	2.78	.45	− .06	− .40	3.10	0.51	− 0.42	− 0.15
EX3 exhibicionism	2.59	.47	.00	− .01	2.72	0.56	− 0.17	− 0.31
EX4 sociability	2.58	.45	− .26	.26	2.92	0.50	− 0.38	− 0.11
NE1 anxiety	2.16	.46	.18	.14	2.16	0.62	0.32	− 0.48
NE2 depression	2.32	.48	.31	.07	2.31	0.59	0.16	− 0.62
NE3 dependence	2.32	.42	− .22	− .22	2.42	0.52	0.22	− 0.35
NE4 low self-esteem	2.21	.56	.06	− .30	2.25	0.67	0.30	− 0.43
Aggressiveness factor	2.14	.37	.24	− .19	2.20	0.44	0.36	− 0.06
Neuroticism factor	2.25	.41	.05	− .15	2.29	0.53	0.25	− 0.47
Sensation seeking factor	2.22	.33	− .04	− .17	2.32	0.41	0.12	0.17
Extraversion factor	2.74	.37	− .21	− .03	3.00	0.40	− 0.33	0.10
Activity factor	2.57	.35	.28	.63	2.68	0.39	− 0.03	0.12

**Table 3:** Reliability of scales per cultures.

	Items with item-total correlation < .10	Thai						Hungarian					
		McDonald’s ω	95% CI lower bound	95% CI upper bound	Cronbach’s α	95% CI lower bound	95% CI upper bound	McDonald’s ω	95% CI lower bound	95% CI upper bound	Cronbach’s α	95% CI lower bound	95% CI upper bound
AG1		.82	.80	.84	.81	.79	.83	.84	.83	.86	.84	.82	.86
AG2		.57	.52	.62	.56	.50	.60	.77	.74	.80	.77	.74	.79
AG2 modified	146	.60	.56	.65	.59	.54	.64	.75	.72	.78	.74	.71	.77
AG3		.75	.72	.77	.75	.72	.77	.88	.87	.89	.87	.86	.89
AG4		.60	.55	.64	.61	.56	.65	.75	.72	.78	.74	.71	.77
SS1		.71	.68	.74	.71	.68	.74	.81	.79	.84	.81	.79	.83
SS2		.57	.52	.62	.56	.51	.61	.73	.70	.76	.73	.70	.76
SS2 modified	147,167	.62	.58	.66	.61	.56	.65	.72	.69	.75	.72	.68	.75
SS3		.55	.50	.60	.51	.45	.56	.79	.77	.82	.79	.77	.81
SS3 modified	12,132, 152	.62	.58	.66	.59	.54	.63	.77	.75	.80	.77	.74	.79
SS4		.68	.65	.72	.67	.64	.71	.73	.70	.76	.73	.70	.76
SS4 modified	17	.73	.70	.76	.73	.69	.76	.73	.70	.76	.73	.69	.76
AC1		.72	.69	.75	.72	.68	.75	.80	.78	.82	.80	.77	.82
AC2		.65	.61	.69	.65	.61	.69	.83	.81	.85	.83	.81	.85
AC2 modified	48	.67	.63	.71	.67	.63	.71	.82	.80	.84	.82	.80	.84
AC3		.44	.39	.50	.44	.38	.50	.62	.58	.67	.62	.58	.66
AC3 modified	13, 53, 113, 133	.61	.56	.65	.58	.52	.62	.69	.65	.72	.67	.63	.71
AC4		.79	.77	.81	.79	.77	.82	.89	.87	.90	.89	.87	.90
EX1		.77	.75	.80	.78	.75	.80	.79	.76	.81	.79	.77	.82
EX2		.73	.70	.76	.72	.69	.75	.83	.81	.85	.83	.81	.85
EX3		.72	.68	.75	.72	.69	.75	.85	.83	.87	.85	.83	.86
EX4		.70	.67	.73	.70	.66	.73	.80	.77	.82	.79	.77	.82
NE1		.74	.72	.77	.74	.71	.77	.88	.86	.89	.87	.86	.89
NE2		.74	.72	.77	.72	.69	.75	.83	.81	.85	.83	.81	.85
NE3		.62	.58	.67	.63	.58	.67	.80	.77	.82	.79	.77	.82
NE4		.84	.82	.86	.84	.82	.85	.91	.90	.92	.91	.90	.92
AG		.89	.87	.90	.88	.87	.90	.93	.92	.94	.93	.92	.93
SS		.75	.72	.78	.81	.79	.83	.90	.89	.91	.90	.89	.91
AC		.82	.80	.84	.83	.81	.85	.88	.87	.89	.89	.87	.90
EX		.89	.88	.90	.89	.88	.90	.92	.91	.93	.92	.91	.93
NE		.91	.91	.92	.91	.90	.92	.95	.95	.96	.95	.95	.96

Scales showed normality-range skewness and kurtosis values. Reliabilities on most scales were adequate, however, in more scales neither Cronbach alpha nor McDonalds’ Omega values reached an adequate level among Thai participants. Lowest reliability was found in AC3 (Restlessness) scale. In scales, which had lower reliability, we excluded some items (see Table 2), in order to reach the marginal 0.6 reliability values. We recalculated scales (both in Hungarian and Thai samples in order to apply the same calculation in both cultures and to have at least marginally adequate reliability values. Also, it must be noted, that main scales all reached Cronbach alphas of 0.80.

### Factor analyses

The factor analyses (principal axis factoring, Varimax rotation, see Table 4.) replicated the original model^10^ except for AC3 Restlessness scale, which loaded more on Sensation Seeking Factor than to Activity factor in both cultures.

**Table 4:** Factor analytic structure in both cultures.

	Hungarian rotated factor matrix^1^					Thai rotated factor matrix					Tucker congruency coefficient^2^		
	1	2	3	4	5	1	2	3	4	5	HU-SP	THAI-SP	HU-THAI
AC1 work compulsion	**.73**	− .02	− .05	.02	.00	**.81**	.00	.07	.00	.14	.99	.96	.98
AC2 general activity	**.59**	− .11	.22	− .13	.20	**.48**	.11	.05	− .02	.39	.97	.96	.88
AC3 restlessness	**.34**	.22	.25	.24	**.42**	**.38**	.34	− .04	.19	**.43**	**.87**	.94	.90
AC4 work energy	**.73**	− .08	.16	− .34	− .29	**.61**	− .18	.32	− .33	− .01	.99	.94	.94
AG1 physical aggression	− .08	**.66**	− .14	− .02	.31	− .12	**.63**	− .17	.25	.21	1.00	.90	.91
AG2 verbal aggression	− .02	**.78**	.11	.07	.24	.06	**.67**	.05	.17	.11	.98	.91	.97
AG3 anger	− .04	**.75**	.08	**.42**	.13	− .11	**.81**	− .13	.27	− .01	.98	.99	.96
AG4 hostility	− .11	**.63**	− .29	**.47**	.18	− .14	**.58**	− .26	**.46**	.20	1.00	.99	.99
EX1 positive emotions	.29	− .13	**.60**	− **.45**	.03	.31	− .24	**.60**	− **.48**	.10	.99	.99	.99
EX2 social warmth	.03	− .09	**.73**	− .20	− .07	.01	− .15	**.71**	− .33	− .13	.99	.96	.98
EX3 exhibitionism	.06	.22	**.58**	− .14	**.38**	.27	.04	**.59**	− .06	**.32**	.97	.93	.90
EX4 sociability	.09	− .01	**.73**	− .15	.33	.09	− .08	**.76**	− .14	.19	.98	1.00	.98
NE1 anxiety	− .03	.26	− .16	**.84**	.02	− .09	.30	− .19	**.77**	.08	.97	.95	.99
NE2 depression	− .15	.15	− .28	**.81**	.15	− .01	.23	− .28	**.74**	.09	.98	.98	.99
NE3 dependence	− .06	.08	− .01	**.82**	− .09	.00	.22	.02	**.69**	.09	.97	**.88**	.95
NE4 low self-esteem	− .08	.01	− .31	**.80**	.03	− .12	.15	− .27	**.83**	.07	.99	.97	.99
SS1 thrill and adventure seeking	.11	.13	− .05	− .16	**.63**	.00	.12	− .01	.03	**.65**	.99	.98	.96
SS2 experience seeking	.07	.09	.06	.09	**.68**	.12	− .03	.09	.09	**.62**	.93	**.84**	.95
SS3 disinhibition	− .11	.24	.18	.10	**.75**	− .04	.18	.19	.11	**.72**	.99	.96	.97
SS4 boredom susceptibility/impulsivity	− .14	.21	.14	.04	**.51**	− **.47**	.27	− .12	.07	**.15**	.93	**.78**	.63

Significant values are in bold.

^1^Extraction Method: Principal Axis Factoring. Rotation Method: Varimax with Kaiser Normalization Rotation converged in 6 iterations.

^2^Pairwise Comparison for Hungarian (HU), Catalan (CAT) and THAI factor structure.

The global Tucker’s coefficient of congruence (TCC) between the two samples was 0.95. The TCC values for facets were below 0.90 only in cases of AC2 (General Activity) and SS4 (Boredom Susceptibility/Impulsivity). When comparing our two samples one by one with the original Spanish validation sample^9^, the global TCC for Hungarian structure was 0.97 and was 0.94 for the Thai one. Regarding the Hungarian sample, AC3 (Restlessness) received the lowest coefficient (0.87). In relation to the Thai sample, NE3 (Dependence: 0.88), SS2 (Experience Seeking: 0.84) and SS4 (Boredom susceptibility/Impulsivity: 0.78) had scores below 0.90.

We have also run a Confirmatory Factor Analysis (see Table 5) with a multi-group analysis for the original structure to test configural invariance, but the fit indices were not adequate (CMIN/df = 12.70; GFI = 0.75; CFI = 0.74; RMSEA = 0.09 [0.09–0.10]).

**Table 5:** Multigroup CFA with configural, metric, scalar and residual invariance test.

Model	CMIN/DF	P	RMR	GFI	AGFI	PGFI	TLI rho2	CFI	RMSEA	LO 90	HI 90	PCLOSE
Configural invariance (equal structure)												
Default model	12.70	.00	.04	.75	.67	.57	.69	.74	.09	.09	.10	.00
Model modest loadings (.30)	8.22	.00	.02	.84	.78	.61	.81	.85	.07	.07	.08	.00
Model modest loadings (.25)	6.25	.00	.02	.88	.82	.59	.86	.90	.06	.06	.07	.00
Model modest loading (.25) with correlated error terms	4.84	.00	.01	.92	.86	.55	.90	.93	.05	.05	.06	.02
Metric invariance (equal measurement weights)	5.30	.00	.03	.89	.85	.63	.89	.91	.06	.05	.06	.00
Scalar invariance (equal item intercepts, structural covariances)	5.47	.00	.03	.89	.84	.65	.88	.90	.06	.06	.06	.00
Residual invariance (equal measurement residuals)	5.79	.00	.03	.86	.83	.69	.87	.89	.06	.06	.06	.00

However, fit indices increased highly when including secondary loadings (based on modest loadings [> 0.25] published by McCrae^6^, (CMIN/df = 4.84, GFI = 0.92, CFI = 0.93, RMSEA = 0.05 [0.05–0.06]). After, we examined metric, scalar and residual invariance, but neither reached satisfactory fit indices.

### Neural network analyses

**Figure 2:**
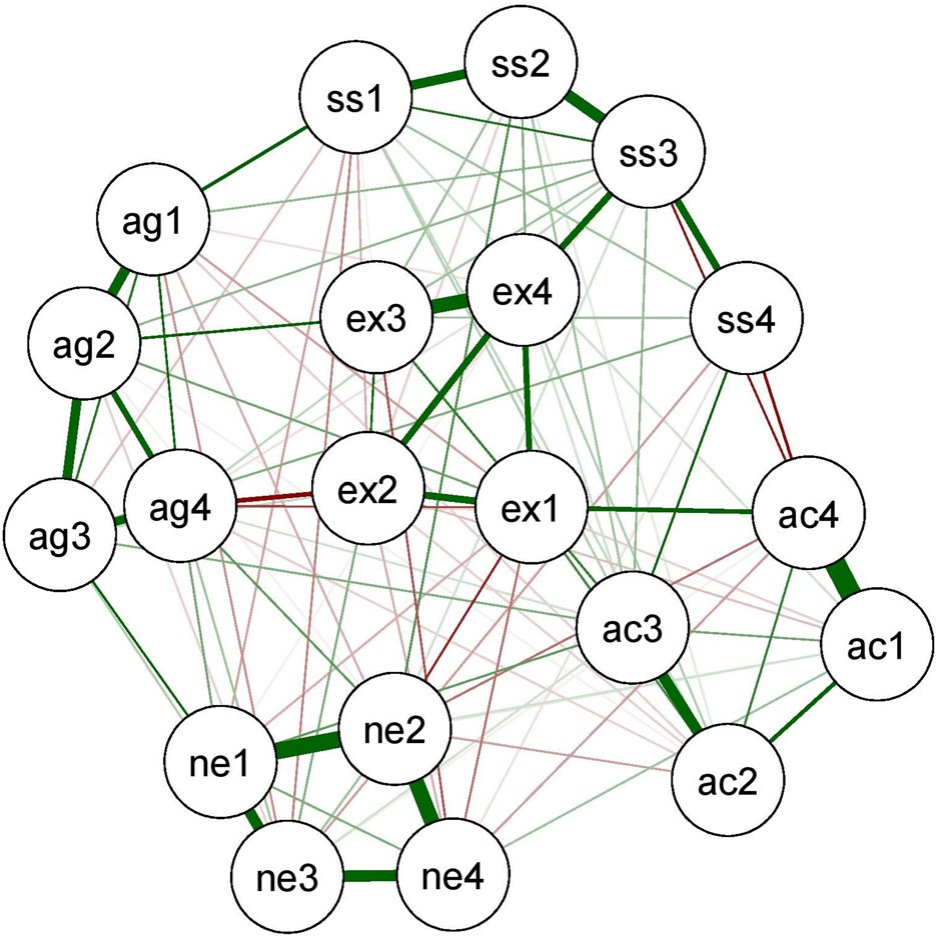
Figure of neural network for Hungarians.

**Figure 3:**
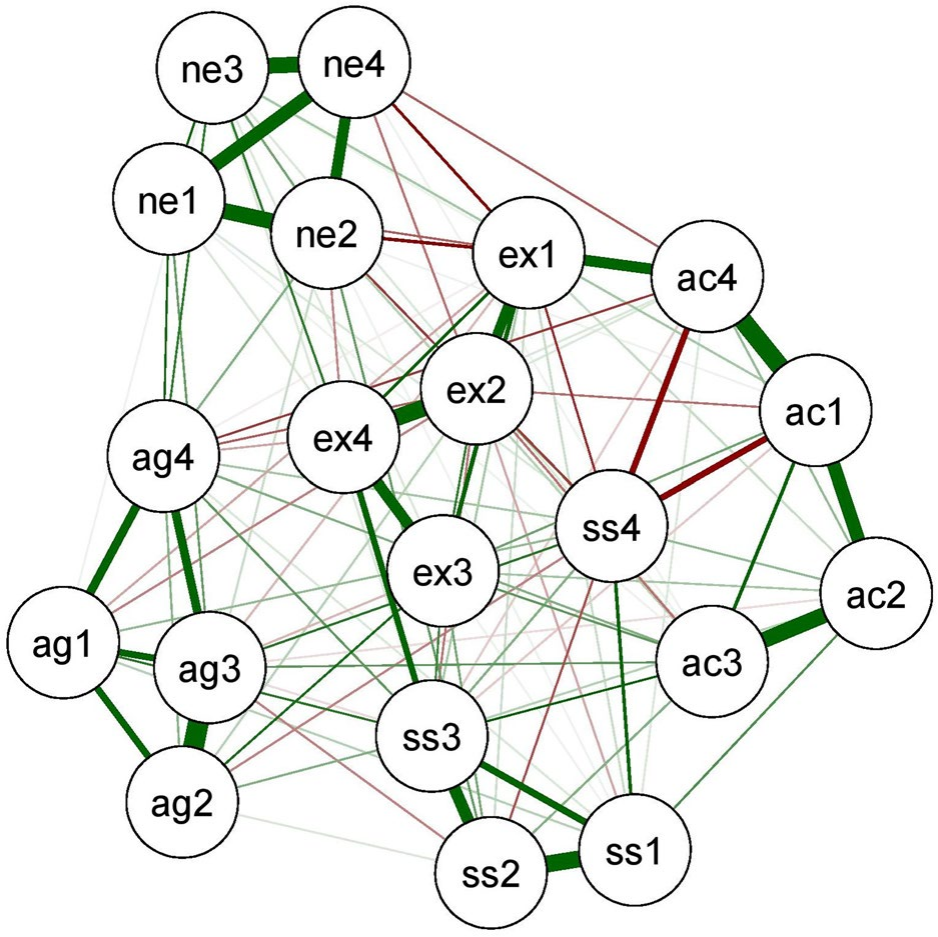
Figure of neural network for Thais.

Neural network analyses (see Figs. 2 and 3) that weights of the connections between these facets differed with at least 0.15 score for the following pairs.

For pairs of AG4 (Hostility)–AG2 (Verbal Aggression), SS3 (Disinhibition)–SS4 (Impulsivity), AC3 (Restlessness)–SS4 (Impulsivity), as well as NE1 (Anxiety)–NE3 (Dependence), weak connections for Thais, definite connections for Hungarians were present. For AC1 (Work compulsion)–SS4 (Impulsivity) pair, no connection for Hungarians, negative connection for Thais were observed. For pairs of EX3 (Exhibitionism)–EX1 (Positive Emotions) and NE1 (Anxiety)–NE4 (Low self-esteem) stronger connections were observed among Thais. Network invariance test (M = 0.20, *p* < 0.000) showed significant difference between the Thai and Hungarian sample. Weights of 53 relations became significantly different, whereas the remaining ones (136 relations) showed cross-cultural invariance. Also, the global strength invariance test (S = 0.26, *p* = 0.83) became nonsignificant.

### Comparison of mean profile scores

Several significant differences arose in the mean facet scores between cultures (see Table 6), however most of them had small effect size (Cohen d smaller than 0.50). Cohen d was above 0.50 only in case of five facets and one factor (EX): Thais scored significantly lower than Hungarians on EX4 Sociability, EX2 Social Warmth, EX1Positive Emotions, AC2 General Activity, SS2 Experience Seeking. Thais had significantly lower level of total aggressiveness (AG), sensation seeking (SS) and activity (AC) as well, although with only small (AG, SS) or medium sized effect size (AC).

**Table 6:** Mean z-score ZKA profiles by culture and significance test.

	Thai		Hungary		Independent samples t-test (if SD equivalence was not met, robust test was performed)			Cohen d
	Mean	SD	Mean	SD	t	df	sig	
Zscore: AG1 physical aggression	− 0.17_a_	0.95	0.17_b_	1.03	− 6.26	1317.00	.00	− .34
Zscore: AG2 verbal aggression	− 0.18_a_	0.88	0.19_b_	1.08	− 6.91	1244.19	.00	− .38
Zscore: AG3 anger	0.04_a_	0.86	− 0.04_a_	1.13	1.37	1208.75	.17	
Zscore: AG4 hostility	0.07_a_	0.95	− 0.08_b_	1.04	2.75	1297.33	.01	.15
Zscore: SS1 thrill and adventure seeking	− 0.08_a_	0.90	0.09_b_	1.09	− 3.06	1254.76	.00	− .17
Zscore: SS2 experience seeking	− 0.24_a_	0.94	0.25_b_	1.00	− 9.27	1317.00	.00	− .51
Zscore: SS3 disinhibition	− 0.01_a_	0.90	0.01_a_	1.09	− 0.23	1253.59	.82	
Zscore: SS4 impulsivity	− 0.04_a_	0.98	0.05_a_	1.02	− 1.63	1317.00	.10	
Zscore: AC1 work compulsion	0.13_a_	0.93	− 0.14_b_	1.05	4.96	1284.27	.00	.27
Zscore: AC2 general activity	− 0.38_a_	0.83	0.40_b_	1.00	− 15.40	1253.98	.00	− .85
Zscore: AC3 restlessness	0.02_a_	0.97	− 0.02_a_	1.03	0.63	1317.00	.53	
Zscore: AC4 work energy	− 0.18_a_	0.91	0.19_b_	1.05	− 6.69	1274.67	.00	− .37
Zscore: EX1 positive emotions	− 0.24_a_	0.99	0.25_b_	.95	− 9.11	1317.00	.00	− .50
Zscore: EX2 social warmth	− 0.31_a_	0.89	0.32_b_	1.01	− 11.96	1285.41	.00	− .66
Zscore: EX3 exhibicionism	− 0.12_a_	0.90	0.13_b_	1.08	− 4.63	1256.72	.00	− .26
Zscore: EX4 sociability	− 0.33_a_	0.89	0.35_b_	.99	− 13.09	1289.08	.00	− .72
Zscore: NE1 anxiety	0.00_a_	0.85	0.00_a_	1.14	− 0.15	1193.38	.88	
Zscore: NE2 depression	0.01_a_	0.89	− 0.01_a_	1.10	0.25	1241.78	.81	
Zscore: NE3 dependence	− 0.10_a_	0.88	0.11_b_	1.10	− 3.85	1236.21	.00	− .21
Zscore: NE4 low self-esteem	− 0.03_a_	0.91	0.04_a_	1.09	− 1.28	1259.19	.20	
Zscore: aggressiveness factor	− 0.07_a_	0.91	0.08_b_	1.08	− 2.77	1264.26	.01	− .15
Zscore: neuroticism factor	− 0.04_a_	0.87	0.04_a_	1.12	− 1.34	1220.08	.18	
Zscore: sensation seeking factor	− 0.13_a_	0.87	0.14_b_	1.10	− 4.87	1230.36	.00	− .27
Zscore: extraversion factor	− 0.31_a_	0.91	0.32_b_	.99	− 12.02	1297.59	.00	− .66
Zscore: activity factor	− 0.15_a_	0.93	0.16_b_	1.04	− 5.79	1289.22	.00	− .32

Values in the same row and subtable not sharing the same subscript are significantly different at *p* < .05 in the two-sided test of equality for column means. Tests are adjusted for all pairwise comparisons within a row of each innermost subtable using the Bonferroni correction.

### Cluster analyses

**Figure 4:**
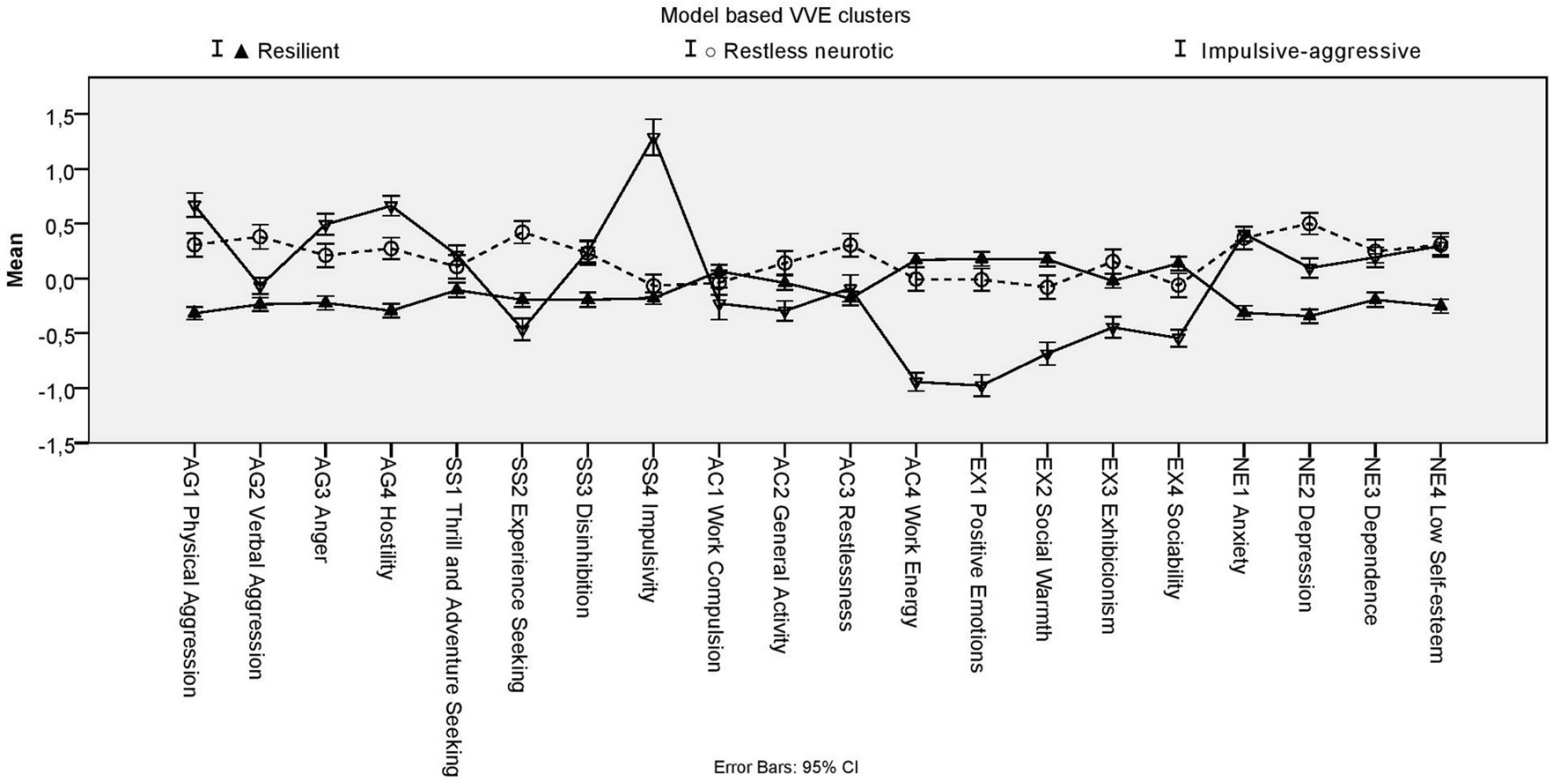
Figure of mean profiles with error bars (95% Confidence Interval) of VVE model based clusters.

We have run probability-based model based clustering and traditional hierarchical cluster analyses with k-means relocation on the combined samples on the 20 facet z scores. Based on model based clustering’s BIC values, solution with three VVE (Ellipsoidal, varying volume and shape and equal orientation) clusters become optimal. One profile (105 Thai and 21 Hungarian individuals) had mean z-scores outside the  − 1 and 1 range (see Fig. 4).

They scored high on SS4 (Impulsivity, z = 1.29), on AG1 (Physical Aggression, z = 0.67) and AG4 (Hostility, z = 0.66), whereas they scored low on AC4 (Work Energy, z = −0.94), EX1 (Positive Emotions, z = −0.98) and EX2 (Social Warmth = −0.54). We named them Introverted impulsive, based on their high scores on impulsivity and low scores on extraversion. The z-scores of the two other profiles were all within −0.50 and 0.50 range. However, one profile had rather higher values (z scores > 0.30) for SS2 (Experience Seeking, z = 0.42), AC3 (Restlessness, z = 0.30), NE1 (Anxiety, z = 0.37), NE2 (Depression, z = 0.50). Therefore we named this profile as Restless neurotic. 171 Thai (36.2%) and 301 Hungarian (63.8%) individuals belonged to this cluster. The third profile scored relatively low on AG1 (Physical aggression, z = −0.32), NE1 (Anxiety, z = −0.31) and NE2 (Depression, z = −0.34), therefore we named them Resilients. 396 Thai (54.9% and 325 Hungarian respondents (45.1%) were assigned to this cluster.

After, we also ran a set of hierarchical clustering, and compared solutions with 2 to 8 clusters. In Table 7, the different cluster adequacy indices can be found for the two to eight-cluster solutions.

**Table 7:** Cluster adequacy indices for hierarchical clustering.

Cluster number	ESS increase	EESS%	Homogenity index mean	Homogenity index min	Homogenity index max
2	1563.08	16.39	1.67	1.52	1.83
3	1060.62	22.32	1.56	1.43	1.87
4	738.48	26.35	1.48	1.36	1.87
5	591.26	29.15	1.42	1.16	1.87
**6**	**425.85**	**31.39**	**1.38**	**0.93**	**1.87**
7	424.77	33.01	1.35	0.93	1.87
8	424.23	34.62	1.32	0.93	1.87
9	321.96	36.23	1.28	0.93	1.78
10	282.12	37.45	1.26	0.93	1.78
11	255.15	38.52	1.24	0.93	1.78
12	251.24	39.49	1.22	0.93	1.78
13	250.23	40.44	1.20	0.75	1.78
14	212.03	41.39	1.19	0.75	1.78
15	191.59	42.19	1.17	0.75	1.78

Significant values are in bold.

If we look at increasement of ESS values, then the rate of increasement comes to a knee point at 6 clusters. It means that the growth rate does not increase from this point. At the 6-cluster solution, minimum score of homogenity index decreases below the level of 1. In Table 8, the different profiles are grouped in order to provide a comparison for the different cluster solutions. We provided names based on a previous study on cross-country ZKA-PQ profiles^34^, in which profile with low EX and high NE was named as 'Overcontrolled', high EX and low NE was named as 'Resilient', high SS and high AG was named as 'Undercontrolled', low SS and low AG was named as 'Reserved', average profile was named 'Ordinary'. (These five profiles are described by previous research^34^.

**Table 8:** Profiles of different clustering solutions with canonical correlation coefficients for discriminating between cultures.

	Canonical correlation coefficient of discriminant analyses for cultures	Resilient profiles	Under-controlled profiles	Over-controlled profiles	Ordinary profiles
Model-based cluster	.27		1: Impulsive-aggressive		1: Rather resilient 2: Rather impulsive
HCL 2	.06	1: Extraverted resilient		1: Overcontrolled neurotic	
HCL 3	.30	1: Extraverted resilient	1: Impulsive	1: Overcontrolled neurotic	
HCL 4	.34	1: Reserved 2: Extraverted resilient	1: Impulsive	1: Overcontrolled neurotic	
HCL 5	.34	1: Reserved 2: Extraverted resilient	1: Aggressive-impulsive 2: Positive Sensation seeker	1: Overcontrolled neurotic	
HCL 6	.37	1: Reserved 2: Extraverted resilient	1: Introverted impulsive 2: Aggressive-impulsive 3: Positive Sensation seeker	1: Overcontrolled neurotic	
HCL 7	.37	1: Reserved 2: Extraverted resilient	1: Introverted impulsive 2: Aggressive-impulsive 3: Positive Sensation seeker	1: Overcontrolled neurotic	1: Ordinary
HCL 8	.34	1: Reserved 2: Extraverted resilient	1: Introverted impulsive 2: Aggressive-impulsive 3: Positive Sensation seeker	1: Overcontrolled neurotic 2: Active neurotic	1: Ordinary

**Figure 5:**
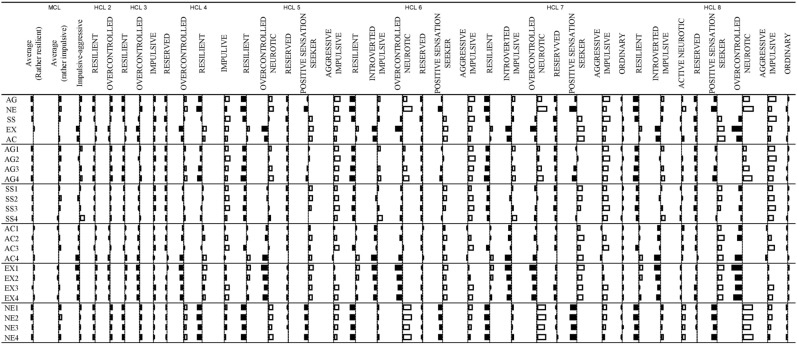
Visualized mean profiles of different clustering solutions.

We can see that the 2-cluster solution provides a resilient and an overcontrolled profile, the 3-cluster solution provides an additional undercontrolled profile (aggressive-impulsive). In the 4-cluster solution there is an additional subtype of resilients (reserved profile). In the 5-cluster solution, a new, more positive undercontrolled profile (positive sensation seeker) is added. In the 6-cluster solution, there is a third undercontolled profile appearing (introverted impulsive). The 7-cluster solution adds only an ordinary profile whereas a new overcontrolled profile (active overcontrolled) appears in 8-cluster solution. Profiles are visualized on Fig. 5.

The cultural differences for cluster assignments were measured by discriminant analyses in order to see how well the cluster assignments could discriminate between cultures. The canonical correlation became the highest for the 6-cluster solution.

This six-cluster solution resulted in two patterns that were more typical among Thai (65.4% and 75.9%), two other patterns that were more typical in Hungary (77% and 68%), and two patterns that were similarly typical among Thai and Hungarian people. The distribution of clusters can be seen in Table 9.

**Table 9:** Cluster distributions by cultures for six-cluster hierarchical cluster analyses solution (with k-means relocation).

		%		N	
		Thai	Hungarian	Thai	Hungarian
Clusters	1: Reserved	**65.4%**	34.6%	206	109
	2: Extraverted resilient	47.8%	52.2%	89	97
	3: Introverted impulsive	**75.9%**	24.1%	186	59
	4: Overcontrolled neurotic	48.0%	52.0%	71	77
	5: Aggressive-impulsive	23.0%	**77.0%**	41	137
	6: Positive sensation seeker	32.0%	**68.0%**	79	168

Significant values are in bold.

**Figure 6:**
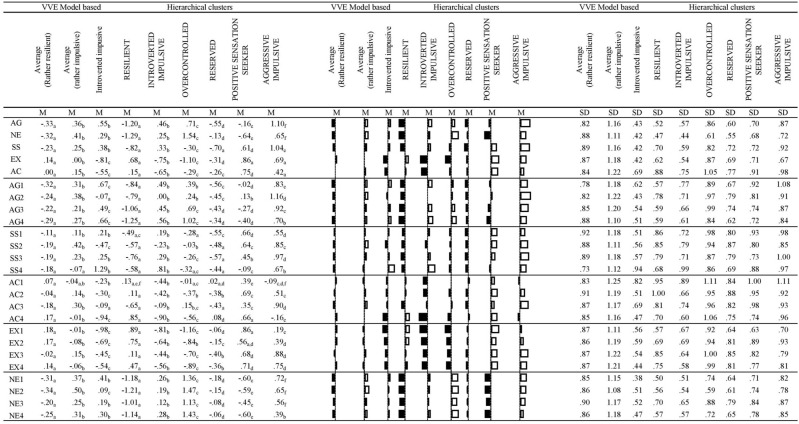
Means (and SD: standard deviations) for Zuckerman-Kuhlman-Aluja Personality Questionnaire’s scale profiles of three-cluster model based cluster-solution and hierarchical six-cluster solution (hierarchical clustering with k-means relocation). Values in the same row and subtable not sharing the same subscript are significantly different at p< .05 in the two-sided test of equality for column means. Tests are adjusted for all pairwise comparisons within a row of each innermost subtable using the Bonferroni correction.

**Figure 7:**
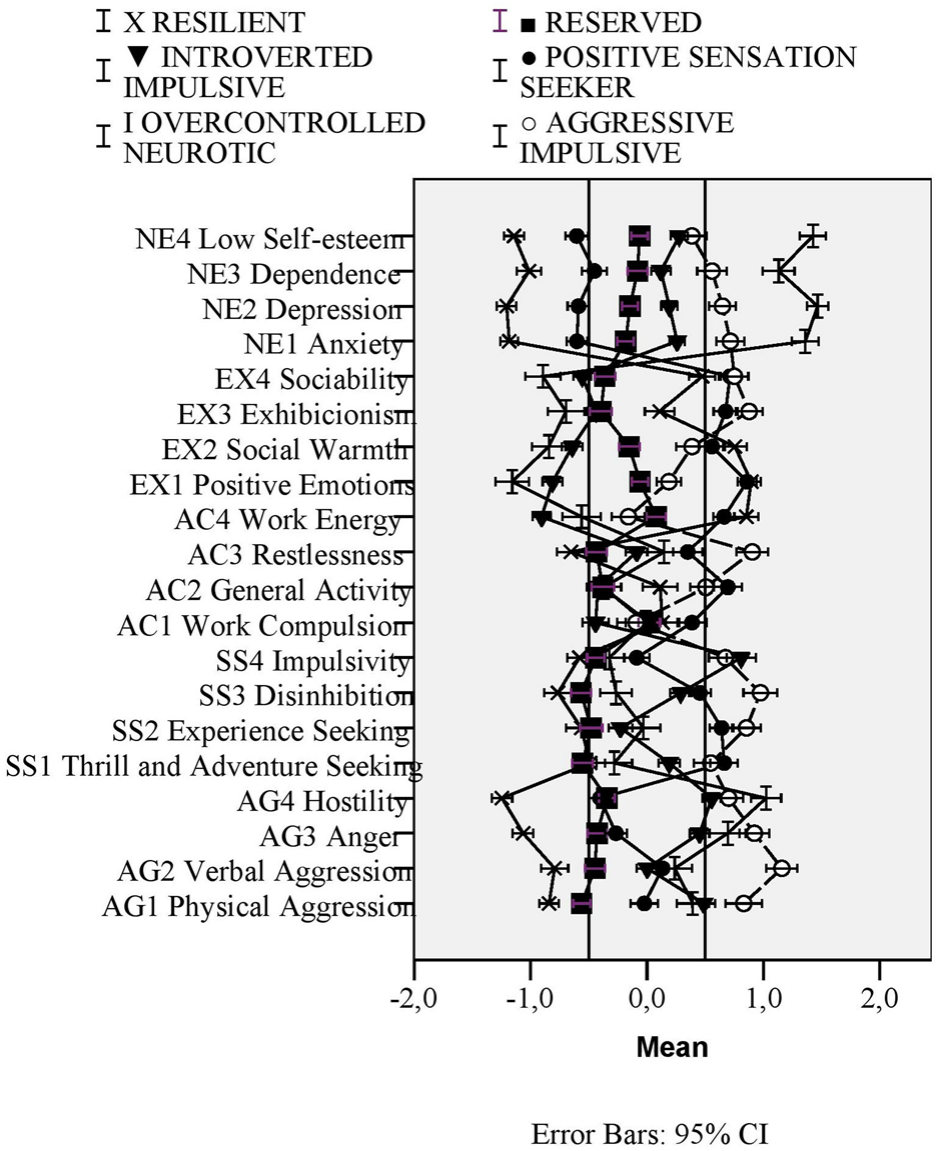
Mean profiles with error bars (95% Confidence Interval) of six-cluster solution (hierarchical clustering with k-means relocation).

Comparing the 6-cluster to the 7-cluster solution, we can conclude that adding a seventh cluster neither increases the discriminative power nor provides a new profile with definite deviations from mean profile. Therefore, we further analyzed the 6-cluster hierarchical cluster solution. The mean profiles ZKA-PQ factor and facet z-scores both for the model-based 3-cluster-solution and for this hierarchical 6-cluster solutions are presented in Fig. 6. According to the results, the 6-cluster solution provides higher deviations from mean profiles (profiles of the 6-clusters are also visualized in Fig. 7).

One cluster (half Thai–half Hungarian) was high in Extraversion (z = 0.68), but low in Aggression (z = −1.20), Neuroticism (z = −1.29) and Sensation seeking (z = −0.82). We named them ‘Resilients’, as they presented similar profile (high EX, low NE) to the Resilients of previous 22-country study of ZKA profiles^61^. The other profile, which we named as ‘Overcontrolled neurotic’, that had around equal number of Thai and Hungarian individuals, was low in Extraversion (z = −1.10), but definitely high in Neuroticism (z = 1.54) and moderately high in Aggression (z = 0.71). In the above-mentioned cross-country study^36^, similar profile was named as ‘Overcontrolled’. The ‘Aggressive-impulsive’ cluster (dominantly Hungarian) was named after their definitely high scores in Aggression factor (z = 1.10 and Impulsive Sensation Seeking factor (z = 1.04). They were similar to cluster of ‘Undercontrolled’ ones in the above-mentioned study^36^. The ‘Positive sensation seeker’ (also dominantly Hungarian) profile was moderately high in Extraversion (z = 0.86), Sensation Seeking (z = 0.61), Activity (z = 0.75) but low in Neuroticism (z = −0.64). The ‘Reserved’ (dominantly Thai) ones were low in Aggressiveness (z = −0.50) and Sensation Seeking (z = −0.70), similarly to ones of ‘Reserved’ cluster of previous cross-country study^36^. The ‘Introverted impulsive’ (other dominantly Thai profile) ones had low scores in Extraversion (z = −0.75), but were impulsive (z = 0.81).

## Discussion

Although our overarching aim was to compare Thai and Hungarian personality profiles of Alternative Five Factor Model dimensions, our study also provides the first validation of ZKA-PQ (Zuckerman–Kuhlman–Aluja Personality Questionnaire, Aluja and colleagues^13^) Thai version. Reliability of the main factors (EX, AG, NE, AC, SS) were adequate. The factor structure of the facets showed similar pattern relative to the results of previous studies for most of the facets^13,15^, except for AC2 (General Activity) and SS4 (Boredom Susceptibility/Impulsivity). The lowest congruence was found in relation to SS4. CFA analyses showed low fit indices, which somewhat increased when allowing secondary loadings, as suggested by Aluja and colleagues^13^. However, only configural invariance was met by also with applying cross-loadings and correlating the error terms. Neural network analyses also confirmed the lack of cross-country invariance for the relations of the facets. The largest difference was found with relation to SS4: it showed relation to Restlessness (AC3) and Disinhibition (SS3) for the Hungarians, but not for the Thais, whereas it showed relation to for Work Compulsion (AC1) for the Thais. These differences can account for lack of factorial invariance, which was observed in relation to SS4.

Although we reported the mean profiles for countries, as well as their comparison by independent samples t-test, the fact that scalar invariance was not met questions the adequacy of comparison. The highest Cohen d was found in case of AC2, however, exactly Tucker Congruency Coefficient for this facet was only 0.88. We also have to note that z-scores for all facets for both countries fell within the average range of z-scores (between −0.50 and 0.50).

However, the differences we found were in congruence with expected results, thus Thai individuals, living in a Buddhist, collectivistic culture, scored lower on Extraversion, Activity and Sensation Seeking. They also scored lower in Aggressiveness, which may be linked to Buddhist traditions and their culture’s low score in Masculinity^30^.

However, comparison of mean profiles remain tentative both due to lack of scalar invariance and lack of between-country deviations from mean profiles. Findings of only weak or negligeable differences in mean personality scores across different cultural groups were also found in other cross-cultural research^15^.

Cluster analytic results, on the other hand, have provided insight into more typical profiles. Clusters of our research results could be linked to previous cross-cultural research on ZKA-PQ clusters^37^, which previously identified resilient, reserved, overcontrolled and undercontrolled types besides the average profile. We additionally identified different subtypes for undercontrolled type: introverted impulsive, aggressive impulsive and positive sensation seeker clusters. Hungarians showed extraverted, whereas Thai showed introverted impulsive patterns as typical undercontrolled profiles.

Out of the six typical profiles, two, rather introverted profiles, were more typically characteristics of Thais: one of reserved, calm, low sensation seekers and another of impulsive aggressive introverted ones. The impulsive Thai profile, on the other hand, is a negative pattern with an inclination toward impulsivity related disorders. The emergence of this introverted – impulsive – aggressive pattern among Thais may be linked to the relatively high prevalence (6.4%) of borderline personality disorder among Thai students^66^ which is a personality disorder with rather low introversion and high impulsivity^67^.

There were two, more typically Hungarian profiles. One was a positive and the other was a negative sensation seeking profile. Although mean profile differences have also indicated the higher sensation seeking level among Hungarians, based on only the mean profile, Hungarians are characterized by higher impulsivity and higher aggressivity as well. Although sensation seeking and its subscale, impulsivity have been linked to a number of dysfunctional behavior^68^, some previous researches have emphasized the need to distinguish between positive and negative patterns of sensation seeking^69,70^. Our cluster analytic results showed that the ‘positive’ sensation seeking type was characterized with low aggression and low neuroticism but high activity. The other, negative pattern of impulsivity among Hungarians was a pattern of high aggression and high neuroticism. Individuals belonging to this cluster could be inclined toward antisocial, narcissistic, borderline or sadistic personality (Cluster B) pathology, according to results of Aluja and colleagues^71^ and Huang and colleagues^72^.

Besides these profiles, we have to note that the ‘Extraverted Resilient’ and ‘Introverted Overcontrolled’ patterns were present in both cultures at similar rates (11–12% for overcontrolled, 13–15% for resilient type). This means that not all Thais could be characterized by an introverted profile, at the same time, neither were all Hungarians highly extraverted.

In sum, mean level group comparisons did not result in reliable comparisons, but examining cluster profiles, more typical profiles, also clinically relevant ones could be unfolded, especially regarding Cluster B pathology.

Finally, we believe that our strategy can be applied to not only cultural group comparisons but to clinical-non-clinical comparison or comparison of differentiation within a clinical group. When examining a clinical group, if we cannot assume the homogenity of the group (which is usually the case), we cannot assume that the mean factor profile is and adequate and satisfactory summation for all individuals within the given clinical group. Clinical practices can be enhanced if patients are treated not as a prototype of a group, but as a person with an individual holistic profile of the psychological, biological and environmental determinants.

## Conclusions

In sum, the Zuckerman–Kuhlman–Aluja Personality Questionnaire measures reliably the five main factors of Alternative Five Factor Model but some facet-level reliabilities were weak, especially within AC factor. There were some cross-cultural congruence in factor structure, but strict invariance was not fulfilled. Comparison of mean profiles remain tentative, but cluster analysis revealed cross-cultural differences in typical profiles: some specific introverted profiles (reserved and introverted impulsive) were more typical to Thai, whereas some specific extraverted profiles (aggressive impulsive and positive sensation seeker) were more typical to Hungarians. However, there were an extraverted resilient and an introverted overcontrolled clusters as well, which were equally present in both cultures.

## Limitations

The main limitation of our study lies in including only one questionnaire, the Zuckerman–Kuhlman–Aluja Personality Questionnaire, therefore besides structural validation, no other validation process (such as concurrent, convergent, predictive and discriminant) could be applied. There were other limitations, such as age-gender and education matching afterwards were applied, only online administration of questionnaire was done (those having no access to computers could not participate), no test–retest measures were applied, no external validation of the clusters were applied.

Future research should incorporate other questionnaires (for concurrent, convergent and discriminant validity) as well as biological measures and apply longitudinal approach in order to test predictive validity of the different personality profiles.

## Data Availability

The datasets generated and/or analysed during the current study are available in the Figshare repository, zka_THAI 672 HU 647_FIGSHARE 22nd July 2023.sav or dataset for ZKA (pakaranhome.com).

## Abbreviations

AC: Activity
AFFM: Alternative five factor model
AG: Aggressiveness
CFA: Confirmatory factor analysis
CFI: Comparative fit index
EX: Extraversion
GFI: Global fit index
NE: Neuroticism
RMSEA: Root mean square error of approximation
SS: Sensation seeking
ZKA-PQ: Zuckerman–Kuhlman–Aluja Personality Questionnaire
